# Reproductive technology’s animal unconscious: multispecies motherhood and humanimal horror

**DOI:** 10.1136/medhum-2025-013322

**Published:** 2025-09-10

**Authors:** Georgia Walton, Dominic O’Key

**Affiliations:** 1 Politics, Philosophy and Religion, Lancaster University, Lancaster, UK; 2 Faculty of English, Cambridge University, Cambridge, UK

**Keywords:** reproductive medicine, Film, cultural studies, pregnancy

## Abstract

Preclinical animal testing has played a critical role within medical history. Yet it remains an underdiscussed topic within the medical humanities. What might happen, then, if we analyse the animal studies of the lab via the method of cultural critique that is animal studies? This essay responds to this question by exploring the roles that animals play, and are made to play, within the technologies for, debates about and narratives of human reproduction. The essay is comparative. First, it focuses on how the Children’s Hospital of Philadelphia (CHOP) represents their experiments with lambs as part of their development of an artificial placenta. Then, it juxtaposes these narratives with two recent horror films that dramatise and hyperbolise the very human–animal relations on which CHOP’s research relies. Both Laura Moss’s *Birth/Rebirth* (2023) and Valdimar Jóhannsson’s *Lamb* (2021) can be read, we suggest, as narratives of multispecies family-making that, in their representation of human reproduction as dependent on the exploitation of animal reproduction, signify and subvert the species hierarchies that attend animal testing. Yet we also wish to track how each of these films, just like CHOP, ultimately figures animals as sacrificial objects. We argue that reading these texts together illuminates the politics of species that undergirds CHOP’s research, its medical humanities critique and the horror genre. More than simply adding an animal studies perspective to the debate about the future of human reproduction, this essay models forms of reading that, by combining the interpretive practices of animal studies and medical humanities, unsettle the ways that animals are positioned as literal and symbolic surrogates for the human.

## Introduction: Silence of the Lambs

 In April 2017, the Children’s Hospital of Philadelphia (CHOP) engaged in a communications campaign to share details of their preclinical experiments in ‘extrauterine support’ (Partridge *et al*. 2017a, 2). Across academic publications, news media interviews and an in-house short film, neonatal doctors working in CHOP’s Center for Fetal Research announced their successful development of a novel extracorporeal device that could reproduce the conditions of later-stage gestation. The device, three years and four prototypes in the making, is an enclosed and sterile system comprising a pumpless oxygenator, replica amniotic fluid, umbilical vascular interface and translucent polyethylene ‘Biobag’ designed to improve the health outcomes of extremely premature infants born before 28 weeks’ gestation (Partridge *et al*. 2017a, 2). It is, in other words, an ‘artificial placenta’ (Partridge *et al*. 2017b, 406).

A frenzy ensued. Legacy news outlets such as CBC News ran interviews with the hospital’s doctors. Digital media companies specialising in short-form clickbait hungrily repackaged CHOP’s footage for their own shareable content (Insider Tech 2017). Beneath these videos, platform users hailed the technology as the end of the abortion debate as we know it, characterised it as a peculiarly creepy evolution of pregnancy, or else humourously pushed the idea of the Biobag towards its hypothetical conclusion: how would it feel if your mother were a plastic bag? In the immediate aftermath, neonatologists penned letters that commended CHOP’s research as an ‘extraordinary development’ in reproductive technologies but cautioned that the pragmatics and ethics of such an experiment were far from resolved (Roberts 2017, 45; Mercurio 2018, 796). Scholars in medical law and bioethics would push these concerns a step further by asking what ‘ectogestation’ means for debates about the ontological definition of the fetus (Kingma and Finn 2020), legal personhood (Romanis 2018) and reproductive rights (Cavaliere 2020; Horn 2020). In the medical humanities, meanwhile, scholars have raised concerns about the technology’s potential impacts on the politics of disability (DeFalco and Dolezal 2021) and analysed its representation in contemporary works of science fiction (Buran 2022).

Yet absent from all these critical interrogations are the very figures central to the technology’s development: the animal test subjects whose ostensible simulation of the conditions of human gestation, and whose representation in the research’s dissemination, constitute the ground on which CHOP’s ostensible medical breakthrough and media attention rests. Indeed, the signature image of the experiment is a small preterm lamb, floating alone in a plastic pouch, its body gently twitching in the fluid. The figure of the lamb stands at the heart of not just the experiment but its descriptions in scientific literature, portrayals by media outlets and reception by readers and viewers. Thus far, however, scholars have taken an interest in the lamb only insofar as it figures as a non-human model whose foetal maturation is assumed to ‘correspond’ (Mercurio 2018, 795) to the prenatal human child. In other words, although scholars have developed persuasive analyses of the Biobag’s political and ethical implications for humans, they have nonetheless overlooked what it means for animals and human–animal relations. As long as we take it for granted that the lamb is indeed a surrogate, then we risk obscuring the ways that CHOP’s research uses lambs as both test subjects and symbolic figures. Put differently, we risk reproducing the fundamentally anthropocentric premise—the ‘fantasy of human exceptionalism’, as Donna Haraway puts it (2008, 11)—of a reproductive experiment that we might otherwise wish to call into question.

This essay, in contrast, suggests that the politics of species is fundamental to both the technologies and representations of the future of human reproduction. Other essays on CHOP’s research tend to begin with the image of the floating lamb only for the animal to then disappear from the analysis that follows as the subject narrows to exclusively human—and, indeed, humanist—concerns. Our aim, though, is to do the opposite: to keep the animal in view.^1^ Preclinical animal testing has played a critical role within medical history. Given this history, what might be gained if we were to analyse these scientific-experimental animal studies via the interdisciplinary method of cultural critique that is animal studies? For it is with animal studies’ interrogation of human–animal relations and ‘the representational problems of animals’ (McHugh 2009, 421), we think, that the medical humanities might make sense of the ways that animals are both materially used and symbolically figured within the technologies for, narratives of, and debates about human reproduction.^2^

Beginning, then, from the premise that the Biobag is a more-than-human device, we wish to bring together the interpretive practices of animal studies and the medical humanities to explore the multispecies politics of human reproduction. Ultimately, this essay seeks to trouble the norms through which the animal becomes figured symbolically as an expendable surrogate. We will pursue this across three parts. In part one, we focus on CHOP’s narration of their experiment. Attending in particular to the ways that they represent the lamb as a model for the human child, we begin by suggesting that their aesthetic repertoire elides not just the individual lambs who have been used in the experiment’s development and the relationships of gestation between ewes and lambs that are broken by these animal trials; it also occludes the networks of industrial animal production and exploitation that make this feasible. Then, we suggest that while the narration of the Biobag troubles the category of the ‘human’, CHOP nevertheless represents its work in ways that restabilise species distinctions. CHOP’s experiment casts the fetus as an ‘alien’ species that recalls both evolutionary lineages of sea-dwelling ancestors and images of cosmic otherness. News networks, moreover, emphasise the science fiction weirdness of the experiment. In response, neonatologists appeal not just to the rationality and necessity of animal research, but also to an ethical and even motherly relationship between the lab and its animal research subjects. We read CHOP’s representation of its experiment as an anticipation of, and attempt to neutralise, the genres of science fiction and horror that are evoked by its interventions into non-human reproduction.

In parts two and three, we turn to two recent films that give form to the very genres that CHOP’s narratives work to nullify. The formally monstrous cinematic offspring of the science fiction and horror genres, Laura Moss’s *Birth/Rebirth* (2023) and Valdimar Jóhannsson’s *Lamb (Dýrið*) (2021) are family dramas in which bereaved mother-protagonists try, with increasing desperation, to revive, recover and replace lost children. Adapting canonical texts and tropes to tell stories of weird parentage, ‘unnatural’ reproduction and hybridised not-quite-human children, *Birth/Rebirth* recasts the *Frankenstein* story via two women’s shared desire to reanimate the dead, while *Lamb* mobilises the transnational aesthetics of folk horror to narrate a couple’s adoption of a humanimal lamb-child. Both of these films can be read, we will suggest, as narratives of multispecies kin-making. Yet we also propose that any affirmative reading must concomitantly account for the ways that these stories represent motherhood as melancholic and monstrous. By staging reproduction’s ‘procreative prerogative’ (Roof 1996, 57) as a form of interspecies domination, these horror films depict animals’ conscription into the logic of human reproduction and family construction, thereby dramatising what CHOP and its response from the medical humanities have thus far overlooked.

In horror studies, it has long been argued that the monstrosity of women characters tends to emerge through plotted conflicts about mothering that allegorise wider contradictions between female autonomy, paternal law and biological reproduction. In Creed’s field-defining theorisation of the ‘monstrous-feminine’ (1993, 3), for example, she locates across horror cinema an abject fictional motherhood that, by threatening and transgressing patriarchal authority, subverts the real burdens that real mothers are made to carry. Horror, in other words, has an uncanny generic affordance to hyperbolise the symbolic condition of motherhood, a condition which, to paraphrase Jacqueline Rose, is constructed by the dominant culture as a paradox: compulsory and expendable. As we will see below, the mothers of *Birth/Rebirth* and *Lamb* do sacrifice themselves for their lost children. Yet although these moments of abnegation exemplify Rose’s argument that mothers remain our favourite sacrificial objects, as disposable as they are indispensable to life (2018, 63), what these two films showcase is horror’s enduring reliance on the logic of sacrifice itself: for in each, the continuations and resolutions of the mother’s plotted attempt to recapture the lost child are established through the narrative sacrifice of animals.

In the canon of horror’s monstrous-feminine figures, mothers are often conceptualised as figures who trouble the boundaries between humanity and animality: reproduction perpetuates human society, while gestation signals the mother’s biology and animality, connoting what Creed calls the monstrous-feminine’s ‘special relationship to the animal world’ (47). Building on a broader feminist concern with the fact that ‘the male mind has always been haunted by the force of the idea of *dependence on a woman for life itself*’, as Adrienne Rich once put it (1995, 11), Creed suggests that the horror genre’s preoccupation with reproductive anxiety ‘speaks to us more about male fears than about female desire or feminine subjectivity’ (1993, 7). *Birth/Rebirth* and *Lamb* play with this humanimal horror of motherhood, framing their characters’ stories as subjective desires played out to their logical ends. But what distinguishes these narratives of intertextual adaptation and multispecies adoption from a wider trend in contemporary cinematic representations of reproduction—whether *The Coffee Table* (2022) or *Immaculate* (2024)—is precisely their narrative investment in human–animal relations. Dwelling thematically and aesthetically on the monstrosity of a grieving motherhood that turns the figure of the animal into a surrogate for the lost object, these films narrate the kinds of interspecies violence that attend human reproduction and novel reproductive technologies.

We contend that reading these texts can illuminate the kinds of institutionalised human sovereignty—the instrumental administration of care and death—that constitute but remain absent from CHOP’s research, while also revealing the ways in which horror cinema itself traffics in animal symbols. If our approach is successful, though, then we also hope to make a specific intervention into the medical humanities. To adapt Fredric Jameson’s terms, we might locate within the medical humanities an ‘animal unconscious’: the politics of species is fundamental to medical history but largely absent from medical humanities study. Paraphrasing Jameson’s critical hermeneutics, we intend to recover the ‘repressed and buried reality’ (Jameson 2002, 4) of those human–animal relations that undergird the research and imaginaries of reproductive futures: the conditions that bring lambs and ewes into the lab, and the forms of mediation that bring only some of these animals into view.

While the medical humanities have hitherto overlooked the ways in which animal lives shape and are shaped by human health concerns, there is an emerging body of work that seeks to address this. Our aim, by building on recent work by the Animal Research Nexus project and the Non-Human Animals in the Medical Humanities Network, is to develop ways of including animals and human–animal relations as primary objects of medical humanities study — if only to ‘produce knowledge that supports the goal of *removing* animals from relationships perceived to be exploitative or harmful to them’ (Giraud and Renelle 2024).^3^ Rather than simply adding an animal studies perspective to the ongoing debate about the future of human reproduction, our essay sets out to put pressure on the often tacit humanisms of the medical humanities as such.

## Part 1: aquatic species

The EXTEND (EXTrauterine Environment for Neonatal Development) research group, who led on the Biobag research, communicated their findings through academic journals, news media interviews and an 8 min short film released on CHOP’s own YouTube channel, which has uploaded over one thousand videos since its launch in 2009. Titled ‘Recreating the womb: an aquatic environment for premature infants’, the promotional documentary presents a humanitarian narrative about precious life in need of urgent rescue and the glimmer of salvation now made newly available and visible through scientific progress. Extreme prematurity, the neonatal doctors state, is the leading cause of infant mortality and morbidity; although conventional modalities of care boost survival rates and health outcomes, those born under 28 weeks’ gestation remain uniquely vulnerable, and it is a tragedy—for parents and doctors alike—to witness a premature baby struggle. For 50 years and more, researchers have tried and failed to develop an artificial placenta; but this new technology has opened up ‘a new hope for premature infants’. This, then, is an archetypal story of triumph against the odds. As one researcher puts it: ‘Never give up, you’ve got to keep going, keep going until it works’ (Children’s Hospital of Philadelphia (CHOP) 2018).

Combining workplace footage, talking-head soundbites and a melodic piano soundtrack, the film’s documentary technique conveys sober factual representation and heartstrings tonal emotion in order to frame its story of context, challenge and optimistic solution. The intensely white colour scheme creates a halo-like glow to the soft focus and brightly lit shots of newborns’ hands and feet as Dr Emily Partridge, a researcher on the team, explains to the camera that babies in the neonatal intensive care unit (NICU), while ‘fascinatingly resilient’, present a ‘real clinical conundrum’ (Children’s Hospital of Philadelphia (CHOP) 2018). As she speaks, an extradiegetic electric beeping sound enters the mix, invoking the sounds of NICU machines and signalling a safety, control and care that mitigates against the unthinkable, dreadful opposite. Balancing sterility and sentiment, the film pitches CHOP’s research not just as a solution to the pathos of premature mortality and morbidity, but also as the alleviator of the anxieties that the NICU represents.

Positioning the BioBag as ‘The Breakthrough’, the film switches from video footage to the animation of a pumping heart visible in the foetal lamb’s chest cavity. When it comes to showing the lamb itself, then, the film shifts away from the documentary style it has used elsewhere, adopting instead its own representational surrogate. These animated segments are accompanied by footage of Dr Alan Flake, Director of the Center for Fetal Research, who explains that lambs are appropriate models because of their already-established role in foetal research. Though the team speaks plainly, without regret, about the use of these animal models, discussion of specific lambs themselves is kept to a minimum; the doctors do not expand on the origins of the lambs, nor on the specifics of how they are used in the experiment. In their corresponding research papers, however, the researchers note that they purchase Suffolk and Hampshire ewes from a United States Department of Agriculture-approved farm. The experiment involves anaesthetising the ewes, performing a laparotomy, extracting the foetal lamb and submerging it in the Biobag’s sterile amnion; the ewes are then killed by pentobarbital sodium injection. The doctors monitor the lamb’s development in the Biobag, and then—if it survives—transition the body from fluid to ventilator, after which it is euthanised and subject to an autopsy (Hornick *et al.* 2019; Cohen *et al*. 2024). The kinds of reproductive autonomy that the Biobag imagines for humans are thus made possible by the denial of autonomy to animal subjects, whose gestational relationships are severed and whose lives are prematurely ended. The video’s omission of these material circumstances is part of the euphemistic mode adopted by the team when discussing the lamb’s role in the experiment in which the word ‘preclinical’ stands in for the animal testing on which it relies. The figure of the lamb is hyper-visible, but lambs and ewes are obscured.

The Biobag’s ostensible success as an experiment, its media traction, *and* its critical response from bioethics and medical humanities are contingent on the simultaneous figuration and concealment of animality. On the one hand, these are nothing other than animal tests, not human ones, and thus the film’s humanising aesthetics work, although uneasily, to institute a metaphoric leap from real animal to imagined human, the extrapolation of exclusively non-human results to future human possibilities. On the other hand, the experiment’s apparent success demonstrates that the prospect of improving human health outcomes necessitates an acknowledgement that the neonate, or ‘preemie’ as researchers often refer to it, is not quite a child, nor an infant, nor even a human. The problem with five decades of artificial womb research, the neonatal doctors say in the film, is that prior research teams had modelled their research on the needs of a premature newborn baby. The trick is to switch perspective, as Dr Marcus Davey points out: ‘Let’s not treat this as a newborn baby, but let’s treat this as a fetus’ (Children’s Hospital of Philadelphia (CHOP) 2018). At the heart of their method and approach is thus a shift in ontological reclassification. Attending to the differences between the vulnerable human ‘infant’ in the air of the incubator and the distinct species of ‘fetus’ that floats in the fluid of the bag (Partridge *et al*. 2017a, 9), the short film configures the premature life form—whether the lamb test subject or future human fetus—as a temporarily aquatic species that must be submerged in warm and sterile fluid, enclosed in the quiet and darkness.

By foregrounding the fetus’s amphibious humanity, CHOP’s short film emphasises not just that biological differences are immanent to the human but that these differences are historically constructed. Yet there is an impulse, here, to shore up a conceptualisation of the human being that has otherwise been unsettled by the research findings. The team explicitly rejects any commentary that positions their experiment as a precursor to a cyborg future of ectogenesis, preferring instead to emphasise the Biobag’s continuity with incubation techniques that have been used in neonatal care since the 19th century (De Bie et al. 2023). They position the technology only as a ‘bridge’ and ‘transition’ (Children’s Hospital of Philadelphia (CHOP) 2018) that will enable the fetus to become infant, that is, to progress from one species to another, from aquatic life to the “dry land” that Partridge refers to in the short film (Children’s Hospital of Philadelphia (CHOP) (2018)). Where the lamb remains neatly categorised as expendable, the apparent non-humanity of the unborn fetus, what Romanis describes as the “gestateling” (2019) is obviated as species hierarchies are reinstalled.

The Biobag is thus imagined and delimited as a vehicle for what Megan H. Glick terms ‘infrahuman’ subjectivity, a ‘position of liminal human speciation created within an anthropocentric frame that disturbs and reestablishes ‘hierarchies of speciation’ (2018, 4). While the experiments disclose that modern conceptualisations of ‘the human’ are fuzzy and provisional, the subject of social categorisations riven by biological difference and configured historically for competing interests, CHOP’s representation of its work symbolically represses, diverts and otherwise channels this knowledge into a biopolitics in which the social boundaries of species orders are reinforced: the lamb’s stated physiological similarity to the gestateling poses problems for the binary between human and animal, yet its unquestioned use and death within the biomedical setting reinstalls the human as the arbiter of what life matters and is or is not grievable. Animal research may be deemed viable because of an indistinction between species, yet it is deemed permissible only because of an ultimate claim to distinction.

The point, as Sophie Lewis writes, is that fetal subjects, as members of a peculiar aquatic species whose humanity is socially constructed but biologically liminal, might demand from us ‘all of the same ethical agonisms of a multispecies companionship’ (2019). The body is not only pluralised by pregnancy, becoming more than one, but also hybridised, becoming more than human. Yet CHOP rebuilds the species boundaries that would preserve extant human–animal relations, legitimising its exercise of human sovereignty over other forms of life. By portraying the lamb only as the ‘classical animal model for fetal surgical intervention’ (CBC News 2017), they naturalise and instrumentalise the animal as useful but ultimately disposable matter. Only this can explain the team’s claims that ‘Our lambs receive everything that they receive in their mothers’ wombs in a very physiologic way’, or as Partridge puts it in the short film: ‘It never fails to strike me what a miracle it is to see this fetus that is clearly not ready to be born enclosed in this fluid space, breathing, swallowing, swimming, dreaming with complete detachment from the placenta and from mom’ (Children’s Hospital of Philadelphia (CHOP) 2018).

Here, then, is the only acknowledgement of the euthanised ewe in CHOP’s self-narration. The personification of the ewe as ‘mom’ appears to finally collapse the binary categories of human and animal, yet this articulation is only possible because of the stated ‘miracle’ of the fetus’s ‘complete detachment’ from its mother, a moment consolidated by the innocent image of it ‘breathing, swallowing, swimming, dreaming’. There is no word here for exactly *how* ewes have been brought into this system. There is no admission that the bag cannot nurse or care in a comparable way to ‘mom’, nor is there an acknowledgement—as Roberts has pointed out—that mammalian gestation is a two-way process of maternal–foetal ‘crosstalk’ (2017, 46), in which pregnant bodies not only give and take from the placenta and fetus, but are also gestated by the fetus. The experiment nurtures and kills the ewe and the lamb, terminating both their lives and any possibility of them having a relationship with one another.

This logic of maternal disembodiment, in which the pregnant body is disarticulated from the fetus, has long been a feature of the visual rhetoric of prenatal biotechnologies. As Nathan Stormer has argued, in the second half of the twentieth century, foetal imaging was leveraged in ways that would ‘habitually position the unborn…outside the mother’s body’ (Stormer 2008, 653). From Lennart Nilsson’s photographs in Life magazine to the later pamphlets of the anti-abortionist right, the unborn was symbolised via sonographs, microscopic photographs and computer-enhanced MRIs in a free, abstract and cosmic infinity. The sublime aesthetics rendered the fetus safely enclosed yet vulnerable to the vast, powerful and mysterious space of an eternal ‘life’ beyond. This visual regime may anticipate the futures of human reproduction that CHOP says it is inventing, but the image it conjures is curiously non-human: for it is a lamb, not a human, that is suspended in liquid. This may still invoke a kind of prenatal sublimity, but it is no longer situated within the infinity of space, but rather the pinched corners of the polythene bag. Where the mid-century foetal image pointed, optimistically if contradictorily, to the possibilities of anonymity in the void, CHOP’s ovine fetus is subject to a deeper regime of surveillance where the ‘miracle’ of life is deferred from animal model to future human recipients.

Today, as members of the EXTEND research team rebrand themselves as Vitara Biomedical in a bid to secure venture capital funding for the device, their website further enshrines the humanist imaginary of the technology.^4^ The role of sheep as test subjects in their experiment is now completely erased. The caesarean section performed on a pregnant ewe, the cannulation of the lamb’s umbilical cord and the submergence of the animal in the bag’s liquid, and the euthanasia and dissection of the lamb—all of this makes possible, but goes unsaid, as the team announces a ‘new era in neonatal care’.^4^

## Part 2: mad scientist princess bitch

Like the creators of the Biobag, Dr Rose Casper (Marin Ireland) understands the fundamental role of the amnion in sustaining new life. As *Birth/Rebirth’s* Victor Frankenstein figure, she finds the procreative capabilities of science to lie not, as they did for her forbear, in the ability to bypass the maternal body, but rather in the harnessing of its productive power. Grieving the loss of her mother and fascinated from an early age with the regenerative capabilities of starfish, Rose has been secretly exploiting her position as a pathologist at a New York hospital to work on what she calls an ‘experimental treatment […] for death’ (Moss 2023) made from the fetal tissue, amniotic fluid and placenta of gestating bodies. Sourcing these materials is at once medically unethical and personally dangerous to Rose’s health, as she plans out cycles of self-impregnation and miscarriage so as to produce the required components of her serum. She finds early success in the revival of a pig whom she names after her late mother, Muriel. But when Rose makes the fateful move of stealing a child’s body from the morgue, which brings her into contact with the child’s single mother Celie Morales (Judy Reyes), it kickstarts a turn of events that will lead to increasingly desperate acts of violence against other gestating bodies. *Birth/Rebirth* thus depicts what CHOP’s short film suppresses: how biomedical experiments made in the name of an imagined future motherhood come at the expense of actually existing gestational relationships.

Rose and Celie work in the same hospital, but the film establishes their difference from the very beginning. Rose is characterised as isolated, cold and self-interested. An unsociable and hard-nosed scientist who analyses cadavers alone in her underground laboratory, Rose is a single, childless, vigilante doctor, played by Ireland as aloof and awkward: a ‘mad scientist princess bitch’, as she’s described at one point in the film. Celie, in contrast, is portrayed as a warm and responsive mother who, as a midwife, works overtime to bring new life into the world. In the opening sequence, the film juxtaposes scenes of Rose in the lab, her hands in open wounds of cadavers, with Celie in the NICU, her finger clasped by a baby in an incubator. Where Rose’s surname, Casper, denotes in this case a rather unfriendly ghostliness, Celie’s is identified by default on the side of morality. If Rose might be said to personify science, Celie embodies medicine. When the two women argue over their methods of care, Rose explodes that ‘this ad hoc triage I’ve been doing, this isn’t science’, to which Celie replies, ‘No, it’s medicine. But you’re not that kind of doctor, are you?’

Indeed, their differences are brought into intimate relation after Rose has reanimated Celie’s deceased daughter, Lila. Neither Rose nor Celie wants to let the undead Lila go. For the former, Lila is first an ideal test subject and later testament to what she calls ‘good science and hard work over time’. For the latter, she is nothing short of a second chance at motherhood. For both, Lila is their raison d’être, and the two women thus begin living together and co-parenting the zombified child: cooking for one another, squabbling over domestic matters and taking it in turns to care for Lila, swapping stories of her development as parents would an infant. If *Birth/Rebirth*’s family drama sees Rose slowly become Lila’s second mother as she gestates the materials for the life-sustaining serum, it also depicts Celie’s increasing abandonment of ethics as she plots to extract the amniotic fluid from a pregnant woman under her care. Just as the forward slash in the film’s title demarcates an ambiguity about the border between life and death, so too does its narrative give us two characters who, beginning as opposites, become increasingly indistinguishable as the film goes on.

*Birth/Rebirth* thus stages a prototypically human drama: how the mother labours to keep the child alive. Yet the film also derives its plot from another familiar story. *Birth/Rebirth* is just one among many works that, since the publication of Mary Shelley’s novel two centuries ago, have turned to *Frankenstein* as a ‘de facto intertext for […] conversations about science, technology and the ethical obligations of creation’ (Wilson-Bates 2024, 29), expressing what Judith Roof terms ‘anxieties about an unauthorized reproduction that challenges proper (ie, paternal) reproductive order and human aegis’ (1996, 10). More specifically, the film’s gender-swapped narrative continues a more recent trend of cinematic adaptations that, as Robert I. Lublin notes, emphasise female subjectivity within the story (2024, 121). *Birth/Rebirth* pushes this gendered reversal further by upending the central conflict of Shelley’s text: Frankenstein’s rejection of the Creature. Indeed, as the characters converge as co-mother figures, the film plays out not what comes after the father’s rejection of the object, but rather what becomes of two mothers’ fanatical devotion to it. If this recasts *Frankenstein’s* scene of artificial creation as a story about the dangers that might arise from loving one’s child too much, and at the expense of other life, then it also evokes Shelley’s own biographical experiences of child loss that many critics have read as a latent element of the novel’s fantasy of reanimation (Mellor 1988; Bennett 1998; Crook 1996).

At the same time, *Birth/Rebirth*’s intertextual adaptation transforms Rose into both creator and creature. Like Victor Frankenstein before her, Rose personifies a callous Promethean overreach into the natural order that dissects and reanimates life at will in the name of scientific progress. Yet just like Frankenstein’s monster, Rose is characterised as a conscientious defier of carnivory; she refuses to eat meat even when recovering from surgery on her cervix. Where Shelley figures Frankenstein as the carnivore and his ‘daemon’ creation as a vegetarian – ‘My food’, the creature says, ‘is not that of man; I do not destroy the lamb and the kid to glut my appetite; acorns and berries afford me sufficient nourishment’ (2003, 147)—*Birth/Rebirth* slides the two figures into one another via the politics of eating and meat production. In this, the film makes visible the latent vegetarianism of Shelley’s novel, which was authored within the context of a radical Romantic social circle that advocated for a vegetarian diet (Shelley 1884). Rose thus embodies three archetypes of horror monstrosity: the mad scientist, the monstrous-feminine and what Emelia Quinn has recently termed the ‘monstrous vegan’—a trope, originating in *Frankenstein*, in which a character’s animal abstinence troubles social orders (2021, 3).

Indeed, *Birth/Rebirth* uses Rose’s veganism to mark her isolation from the human community. A maladaptation to the world and a withdrawal from reality, veganism is presented as a stubborn ethical stance towards animals within the context of a life of continual ethical infringements against humans. Celie is horrified when she discovers footage of Rose using her own mother’s body as a test subject for her early reanimation experiments. ‘Was she an organ donor too?’, Celie asks. When Rose replies affirmatively, recognising the irony of the question but submitting to answer it sincerely, Celie counters with yet more sarcasm: ‘Oh, good, I wouldn’t want you to do something unethical, like eat a ham sandwich’. Rose’s veganism is thus interpreted by Celie as hypocrisy, a perverse surfeit of concern for animals yet indifference towards humans. For Celie, then, Rose exhibits both Frankenstein’s all-too-human hubris and the monster’s all-too-inhuman misanthropy.

Yet it is through the figure of the animal that Rose develops her own kind of motherly relation. *Birth/Rebirth* incorporates the undead pig as a key figure in its story of human reproduction. Muriel functions for Rose as a test subject, but named after Rose’s mother, the pig also stands as both Rose’s replacement parent and her only child. Unlike the lambs in the Biobag, Muriel becomes integrated into the family unit. In the film’s second act, Celie and Rose settle into a routine, jointly caring for Muriel and Lila as well as one another. In this way, the pig doesn’t just stand in for, but actually becomes a child, with Celie referring to Rose as Muriel’s ‘mommy’. What’s more, Lila and Muriel come to be represented as having more in common with one another than they do with their human mothers. In Shelley’s novel, the creature is a product of the abattoir and charnel house, a hybrid body composed out of human and animal remains. Lila, too, is increasingly aligned with animals. Before her death, she is characterised as a child fascinated by spiders. When she later wakes up, now undead, she is emotionally dependent on her soft toy duck and captivated by a fictional children’s TV show called *The Rescue Birds*, in which three brightly coloured songbirds help other animals in need. Based on a real series, *Wonder Pets!*, which ran from 2009 to 2016 on Nickelodeon before a 2024 reboot for Apple TV+, the characters in *Rescue Birds* repeatedly sing ‘We’ll make sure every animal is okay, that’s what we do, we’re the rescue birds’. This catchy song chimes throughout the film, pointing to the kinds of ‘rescue’ that Rose and Celie’s plot entails and thus ironising the forms of anthropocentric saviourism that animate the imaginaries of human reproduction.

Yet this formation of a multispecies family cannot hold. During a period of mounting tension and narrative suspense after several failed attempts to remake the life-giving serum, Lila crawls out of bed and, without warning, stabs Muriel with the base of her intravenous drip. Usurping Muriel’s role as Rose’s mother-daughter, Lila establishes her separation from and dominance over the animal. Narratively, Muriel’s death reproduces a well-worn generic trope within horror cinema: the putting to death of the animal, sacrificed on the altar of plot and family. From *Halloween* (1978) to *Funny Games* (2007) and indeed to *Lamb*—as we will see shortly—animals are often killed in the first act of the horror story so as to signal a threat, typically to the nuclear family. The death of an animal, especially the family pet, is plotted into the text as a harbinger of the ‘real’ violence to come. In defaulting to this recognisable trope, *Birth/Rebirth* makes a faltering attempt to reassert an unsettled species boundary. The use of such a common narrative formulation suggests the fragility of the boundary between Lila and Muriel; only by turning to a predictable narrative arc does the film reinstate Lila as a proper human child. Muriel’s death demonstrates the undead child’s monstrosity, but by having Lila kill the pig, the film solidifies its narrative commitment to humanist concerns: what does a human mother owe to the sick child?

In other words, Muriel’s death is a plot device. Functioning narratively as the gateway to Celie’s monstrous actions in the film’s climax, it is only through this scene of slaughter, after Rose has conducted an autopsy on Muriel, that she will realise the need to use her own bone marrow to keep Lila undead. And once Muriel disappears, so too does Rose’s veganism. As Rose recovers from donating her bone marrow, Celie cooks her what looks to be a pork steak. ‘This is meat’, Rose says, ‘I’m not going to eat this’. But eat it she does, ingesting her own surrogate child. This moment signals Rose’s increasing desire to save Lila, a commitment to the human family that requires abandoning her attachments to the animal as both her mother and daughter. With Muriel gone, Rose’s motivations suddenly shift from years of dietary veganism and painstaking unsentimental scientific experimentation to the maternal devotion to the preservation of the zombie child. Recalling the kinds of proanimal thought that circulated in Shelley’s circle, in which the ‘bloody juices‘ of uncooked meat are experienced as ‘raw horror’ (Shelley 1884, 13), made palatable only by Prometheus’ theft of fire from the Gods, the film enacts the ways in which scientific ambition cloaked in humanist concerns neutralises the visceral violence of domination over animals. The film may therefore share with CHOP’s self-narration a sacrificial structure in which animals become surrogates for lost children, but here the animal begins as part of the family until it is violently cast aside. In *Birth/Rebirth*, then, the death of the pig is offered for the narrative completion of a *Frankenstein* adaptation.

## Part 3: María had a little lamb

Set on a remote Icelandic farm surrounded by snow-capped mountains, *Lamb* tells the story of a sheep-farming husband and wife who engage in an unexpected act of interspecies kin-making. The film begins at Christmas. María (Noomi Rapace) and Ingvar (Hilmir Snær Guðnason) work in the barn, assisting their pregnant ewes during the lambing season. The couple is childless and lives together in almost complete silence. When the film’s first scene of dialogue eventually arrives, over 10 min in, its function is to confirm that their childlessness and gloomy quiet are connected as cause and effect. ‘They’re saying time travel is possible now’, Ingvar announces at the dinner table (Jóhannsson 2021). While he confesses that he is not in any hurry to visit the future, María’s halting reply conveys that her attention is fixed elsewhere: ‘I expect it’ll be just as possible… to go back’. Back in time, as the film will disclose, means back to a time before silence and grief, a time in which María was raising her firstborn child, Ada. It is within this context of parental bereavement—expressed by the film’s atmosphere of disquiet and muteness of sound and colour—that the couple will decide to carry one of the newborn lambs out from the barn and into the home.

At least in outline, then, *Lamb* stages a drama of multispecies family-making that disrupts the species logics of the Biobag experiment. There, lambs are sacrificed as test subjects who model possible futures of human reproduction. Here, though, where human reproduction has faltered, where the past is lost and the future seems unthinkable, María and Ingvar commit to caring for what is already here in the present. As the couple devote themselves to their silent, grass-eating adoptee, the film gestures to modes of caring relationality that transcend the ossified species hierarchies implicit within CHOP’s animal research. María’s adoption of the lamb, to adopt the words of Haraway, could thus be read as an act of making kin, not babies (2016, 102). Yet *Lamb* is still a horror film. And this is no ordinary lamb. Indeed, when the couple coax the newborn lamb out from its mother, the viewer comes to assume that María and Ingvar have witnessed the birth of a rather unusual sheep: a chimaera with lamb’s head and human child’s body. As their wordless exchange of gazes turns from confusion into pity and then into longing, María finds the lamb’s humanimality calling out to her. Yet as María names the creature after her lost child, Ada, the film signals that this tale of melancholic motherhood will end badly, as her opening act of object replacement is recast analeptically as a shortcut through grief. The possibilities for an affirmative reading are frustrated by the film’s commitment to the transnational master codes of the genre: what is first represented as multispecies adoption is thus revealed to be an abduction.

Indeed, when they have taken the lamb into their care, the film depicts what CHOP’s narration of the Biobag occludes: the bond between ewe and lamb, and hence the individual and collective desires of animal subjects. *Lamb* communicates this most directly through its contrast, in its first half, by depicting the ewe as a maternal figure. After María has begun feeding the lamb, the film cuts to the barn, where the lamb’s mother paces around her confined enclosure, bleating as she manically twists and turns. When María next visits the barn, the ewe looks up into her eyes, baahing repeatedly until María turns away in frustration. Later, the ewe will escape her pen and trot towards the house; standing under the window where the lamb sleeps, she plaintively bleats. As María commands the ewe to return to the barn, the film cuts to inside the home where Ada watches out the window, calling out in response. All of this culminates in María’s portentous decision to kill the ewe. The sequence starts with María’s nightmare: ewes and rams stare at her, as if peering into her soul. A stylised low-light shot catches the animals’ yellow iridescent eye-shine as a kind of haunting. She wakes in the early morning. It is raining outside. The sound of sheep bleats is no longer background noise of the farm, but is rather heard as a complaint, a judgement on her kidnapping that she can no longer face. The lamb’s mother is outside the window once again. She shoots the ewe without pause, dragging the body away by the horns and burying it in an unmarked patch of dirt—a contrast to the grave of her firstborn child, Ada, that as viewers will see later in the film reads ‘angel on earth, angel in heaven’ [*engill á jörðu – engill á himni*]. María’s multispecies motherhood is thus shown to be founded not just on scenes of violence—the separation of mother from ewe, the killing of the ewe—but also on the denial of the animal’s desires.

Within María’s Christian worldview, represented principally through the sign of the cross that she performs at her child’s graveside, it is perhaps no contradiction or even sin to have killed the ewe. Indeed, the couple think that they are the protagonists of a peculiar restaging of the Christian nativity: as Radio Reykjavik plays *In Dulci Jubilo*, a miracle child is born onto the hay-sheeted floor of a stable, surrounded by shepherds and their flocks; María, appropriately named, will become the hybrid humanoid’s mother, determining that this newborn, as a holy gift, signifies for her and Ingvar a ‘new beginning’. The lamb, a figure of purity and innocence, personifies what Jesus embodied metaphorically: it is the sacrificial lamb of God who, to paraphrase the Gospel of John, takes away the sins of the world. For María, then, the lamb promises to redeem the traumas of the past. Ada is the second coming. Glimpsing the possibility of salvation, she cradles the creature in her arms, thereby taking the lamb out of the animal herd and into the human family.

Yet María and Ingvar are not the characters of a story of special providence and redemption, as the film’s viewers already know. *Lamb* begins with its camera staring out to greyness, a snowstorm scene of low visibility and loud wind gusts. A huddle of black figures emerges from the distance, a herd of wild ponies pressed together for warmth. As the camera advances towards them, we hear a snow-crunch step and a slow, heavy breath. This conveys that what we are watching is not just a traditional dolly tracking shot, used to establish location and mood, but rather the perspective of an unknown life force that is tracking the ponies. An ominous droning sound enters the mix as the ponies dash off to the left and down the hillside, whinnying in fear. The camera, which has now fully assumed the perspective of the anonymous figure, progresses towards a faint light in the distance. The film then cuts to the exterior of an icicle-frosted wooden barn, a symmetrically composed shot in which a horned sheep stands right at the centre, looking out of a window and towards the storm and camera. Inside, the herd nervously watches from their paddocks as the barn door is pulled open. The figure stands shadowed in the entranceway. The ewes scramble to escape. One of them is chosen. After, the ewe limps onto the gangway and drops onto its side, panting heavily in exhaustion.

Thus, unlike in *Birth/Rebirth,* in which men are almost entirely absent, from its start *Lamb*’s plot turns on the question of fatherhood and patriarchal violence. There is in fact a suggestion, in this opening scene, that Ingvar is responsible for bestialising the ewe, an act of paternal desperation that would make him Ada’s true father—without María’s knowledge. Steven Swarbrick and Jean-Thomas Tremblay claim as much in their reading of the film, arguing that the couple’s ‘repression of sodomy’, as the explanation of Ada’s humanimality, is integral to their guiding ‘fantasies of order and progress that, here, take the form of marital healing’ (2024,113). Yet as Swarbrick and Tremblay concede, *Lamb* is primarily the story of a different kind of transgression, in which the characters’ adoption is portrayed as an overreach into nature that must be punished. In this way, the film’s opening sequence functions less as the teasing of bestiality and more the simple utilisation of dramatic irony, as it communicates to viewers that which the couple are not privy to: the humanoid lamb’s real origin, that Ada is therefore not quite the ‘gift’ that María thinks they are, and that there will be repercussions for their child abduction.

For *Lamb* is, after all, a ‘nature bites back’ narrative. And retribution arrives in the shape of a Pan-like humanoid ram. Just as María shot the ewe with impunity, so the ram shoots Ingvar, turning the farmer’s rifle onto the farmer. A moment of explosive violence that becomes the film’s final rupture of its otherwise steady silence, the couple’s fantasy of miraculous redemption is shattered as nature gets even. The ram, the film implies, is Ada’s biological father, the fertile doppelgänger of Ingvar’s bereaved impotence. Coming to reclaim what’s his, he punishes the couple for disobeying the established boundaries of the species. While in the New Testament the lamb signifies God’s gift, that of his only son, in the Old Testament’s Binding of Isaac, the ram comes to replace, and thus save, the human child offered by Isaac as a sacrifice. In *Lamb*, the humanimal ram retaliates against his conscription as the symbol of paternal sacrifice, enacting the retribution of Nature. Cutting against the film’s Christian framings, the ram embodies the ‘folk’ in the film’s generic adoption of folk horror aesthetics. He is a symbol of the old ways, a residual paganism that exists only on the peripheries of modernity, a subterranean force that reasserts itself occasionally yet powerfully. *Lamb* codes this final action as a rebalancing of nature and culture, one that completes its narrative arc as a cautionary tale about hubristic overextensions into nature. As the ram takes the lamb by the hand, the film closes with María looking on, powerless to prevent the loss of her second Ada. The camera cuts to show the humanimal lamb’s face. Is there fear in Ada’s eyes? Do we hear whimpering? Are we therefore meant to think that the lamb is better off with María after all, who, in spite of the violence of the initial child abduction, will continue to care for and socialise the creature into humanity? Or does the film ask its audience to do what María could not: to mourn, accept and let go? Demanding in its denouement that we give up the quietly charismatic infant character to whom we have grown attached, *Lamb* ultimately foregrounds those non-human desires that cut against the anthropocentric fantasy of dominion. María and Ingvar are mere shepherds, the film announces; the humanimal ram is the malevolent god of shepherding.

This is not to say, though, that *Lamb* constructs a mythic binary between an exploitative humanity and an innocent nature. For the ram is, of course, an impure, beastly and violent subject, a humanimal outcast in its own right. The ram’s future, with or without Ada, is as unknown to the viewer as is its past; its position amidst the rural landscape is indeed as marginal and lonely as María’s. Thus, the lamb-child ends the film caught between two worlds: the lamb could stay with María, the adopted human mother who has slaughtered their biological mother, or go with the biological father who has raped their mother. But each, the film suggests, is structured by forms of violence. Although for the film’s global distribution the title became *Lamb*, the original Icelandic, *Dýrið*, simply connotes ‘animal’ or ‘beast’. By signalling the thematics of beastliness, the original title foreshadows the film’s dramatisation of a conflict between social belonging and biological ancestry, played out through these final scenes of beastly violence.

*Lamb*’s refusal of a Manichean dichotomy between humanity’s dominion and nature idolatry evokes the species politics of the Biobag itself, in which—absent the abolition of industrial animal production—there are no easily realisable futures for the experiment’s premature lamb test subjects. We say ‘absent the abolition of industrial animal production’ because the more fundamental horror that *Lamb*’s story registers is, in fact, the terror of sheep farming itself. When, for example, Ingvar is taken to bed blind drunk, Ada rests on his chest as he sleeps, gazing up from the bed towards a landscape painting that hangs on the wall. The camera, momentarily adopting the lamb’s perspective, stabilises on the picture’s content: sheep farmers, just like María and Ingvar, leading their herd across a mountain side. As the camera zooms in, extradiegetic bleats and baahs enter the sound mix to denote the call of the wild. Alerting us to an authentic species-being of which Ada is deprived in the social settings of the family home, the sheep call suggests that, if only Ada were a purebred, not a hybrid, they would be out there in the fields. Yet as the soundtrack grows more ominous—a droning minor note, the sound of human shouting, sheepdog barking—the painting is recast as a scene of brutal domestication, coercion and unfreedom. It is as if Ada recognises in this moment the kinds of anthropocentric aesthetics that heroise human efforts to master nature. The pastoral painting offers a story of human endeavour against the elements that, while full of animal life, naturalises the sheep as mere instruments for human use. Whether in the house or out in the fields, Ada would still be subjugated to social control.

In fact, while Ada might have been pulled out of the animalised herd and into humanised individuality, the rest of the sheep live a life that is all but determined by the biopolitics of agricultural production: made to live, made to die; cared for, even loved, but only insofar as their bodies can be later rendered into meat through production, exchange and consumption. As the film shows, Ingvar and María work across the seasons to delimit the autonomy, health, reproduction and death of the herd. They control breeding habits, squeeze mammary glands, insert their hands into ewes’ vaginas, dehorn young lambs and tag their ears. They bend every ewe–lamb relationship to ultimately commercial ends. This is the norm on which the film’s act of interspecies adoption, as abduction, rests. Indeed, the only thing distinguishing the fate of Ada’s mother from the rest of the herd is that her slaughter is personal; her execution is extricated from the impersonal systems of commodification that attend the couple’s raising, killing and selling of sheep. For every one unnamed mother ewe, there are tens more sheep in the barn and on the fields. None are grievable.

‘Meat animals’, Gabriel Rosenberg writes, are forms of ‘life opened to sex, but life that cannot be raped — flesh that can be touched but that cannot be violated’ (2017, 499). What counts, then, as a horrifying violation? As we have seen, the film stages the humanoid ram’s retaliation to the couple’s individual act of kidnapping. But ultimately, it does not determine that sheep farming—a structural form of animal abduction—is horrifying enough for nature to take any form of revenge. The film’s ending dishes out, as Swarbrick and Tremblay’s analysis shows, the punishment for bestiality as illicit sexual contact between species. However, neither the film nor their reading accounts for the legally sanctioned interspecies sexual contact that is animal husbandry as such. Although *Lamb* has Ingvar and Maria claim Ada as their child, what the film cannot see is that *all* of their sheep are already their children.

## Conclusion

The figure of the animal, we have argued, stands at the limit point for the representations of and debates about the future of human reproduction. Where CHOP’s humanitarian rescue narrative neutralises the horror of animal production through a sentimental appeal to multispecies mothering, the medical humanities have adopted a humanist framing that has by and large ignored the reproductive technology’s politics of species. By overlooking the conditions that render the animal an instrumental model for biomedical research, both the EXTEND group’s self-presentation and the critical response from the medical humanities imagine a reproductive future for humans premised on the reproductive governance of other animals’ lives. Ultimately, therefore, both presuppose an impunity with which biomedicine can use animals as legitimate material and symbolic surrogates, thereby licensing what Derrida has described as the animal’s ‘noncriminal putting to deat’ (1992, 278).

In contrast, *Birth/Rebirth* and *Lamb* adopt the family drama in order to confront this horror head on. Plotting animals’ conscription into the family as replacements for lost loved ones, the films underscore the ways in which faltering human relationships seek repair through animal surrogates. And yet, while both films expose the kinds of human–animal horrors, the repressed and buried reality, of CHOP’s and the medical humanities’ humanism, they also ultimately use their animal figures as sacrificial objects: in one, the pig is made to stand not just as the literal test subject of Dr Casper’s reanimation experiments, but as the symbolic oblation to its *Frankenstein* adaptation, put to death in service of the film’s denouement; in the other, the film’s nature bites back narrative casts multispecies adoption as an abduction that must be punished as anthropocentric hubris, yet its plot suppresses the horror of animal husbandry itself as an institutionalised practice of animal capture.

Taking human–animal relations as its primary object of study, this essay has sought to model how the interpretive practices of animal studies might be brought together with medical humanities approaches. We argue that animals should be considered serious objects of study for the medical humanities—not to further objectify them as surrogates for the human, but so as to consider each animal as the subject of a life.

## Data Availability

All data relevant to the study are included in the article or uploaded as supplemental information.

## Footnotes

In fact, this is why we began the essay without foregrounding the figure of the Lamb. By taking care with our description of the animal’s hypervisible role in the experiment, we wish to question the ways that other works of scholarship have leveraged the lamb only for rhetorical effect.

For more on animal studies and what Robert McKay terms its conscientious attendance to ‘the representational complexity of cultural imaginings of animals’ (2014, 637), see Wolfe (2003) and O’Key (2022).

Another project recently funded by the Wellcome Trust, ‘Multispecies Mutualisms,’ also sets out to explore the effect of multispecies relations on health.

Indeed, as Vitara Biomedical, some members of the research team have further wagered the humanitarian aspects of their work, citing WHO reports in order to position the technology as an urgent response to the ‘global issue’ of extreme prematurity. Yet, despite the fact that preterm births occur at the highest rates in the Global South, their commercialisation is situated squarely in an American context in which the raising of equity financing further consolidates the dominance of elite private healthcare.
